# A Promising Modified Procedure for Upper Eyelid Retraction-Associated Graves’ Ophthalmopathy: Transconjunctival Lateral Levator Aponeurectomy

**Published:** 2017

**Authors:** Fatima KHATAVI, Kobra NASROLLAHI, Alireza ZANDI, Maryam PANAHI, Mahshid MORTAZAVI, Mohsen POURAZIZI, Behzad RANJBAR-OMIDI

**Affiliations:** 1 Isfahan Eye Research Center, Department of Ophthalmology, Isfahan University of Medical Sciences, Isfahan, Iran; 2 Department of Ophthalmology, Isfahan University of Medical Sciences, Isfahan, Iran; 3 Cancer Research Center, Cancer Institute, Tehran University of Medical Sciences, Tehran, Iran.

**Keywords:** Levator Aponeurectomy, Lid Retraction, Graves’ Ophthalmopathy, Thyroid Eye Disease

## Abstract

Upper eyelid retraction is a characteristic feature of thyroid eye disease, including Graves’ orbitopathy. In this study, a new surgical technique for correction of lid retraction secondary to Graves’ orbitopathy is described. Sixteen eyelids of patients older than 18 years old underwent surgical correction for moderate to severe lid retraction secondary to Graves’ orbitopathy. In this procedure, levator aponeurectomy was performed via a transconjunctival approach. Upper marginal reflex distance (MRD1) was measured before the surgery and at 1 week, 3 months, and 6 months after the surgery. MRD1 was reduced significantly from preoperatively (mean: 7.84 mm) to 1 week after the surgery (mean: 3.59 mm) (P < 0.001). Three and six months after surgery, mean MRD1 was 5.09 mm and 5.10 mm, respectively, showing that lid retraction was improved significantly (P < 0.001). Lateral levator aponeurectomy via the transconjunctival approach is a simple, scar-less, quick procedure that has optimal stable outcome.

## INTRODUCTION

Graves’ ophthalmopathy is an organ-specific autoimmune disorder that leads to inflammatory swelling and lymphocytic infiltration in the orbit, causing fibrosis and mucopolysaccharide deposition in the orbital connective tissue. Lid retraction, lid margin malposition, proptosis, limitation of extraocular muscle motility, optic nerve compression, chemosis, blood vessel congestion, and corneal exposure are typical orbital signs and symptoms of the disease [1-3]. Upper eyelid retraction is a characteristic feature of thyroid eye disease (TED) [4-6]. Lid retraction is defined as an abnormally high positioned lid in the affected eye in primary gaze [7, 8]. This condition can cause exposure keratopathy and its disturbing symptoms; it also results in staring faces, aggressive appearance, and limited scope for cosmetic surgery [5, 6, 9]. The mechanism of upper eyelid retraction can be secondary to levator muscle contracture due to inflammation and fibrosis. Lid retraction may be related to the degree of associated proptosis, which may act as a wedge [5, 10, 11]. Another possible mechanism is hyperaction of Müller’s muscle caused by sympathetic enhancement due to high level of thyroid hormones [11, 12]. Treatment of Graves’ orbitopathy is surgical and non-surgical. Non-surgical management of Graves’ orbitopathy includes anti-inflammatory drugs, which, however, may not result in complete resolution and symptom relief. Orbital decompression, correction of squinted eye, lid lengthening, and blepharoplasty are steps of the surgical approach to Graves’ orbitopathy. Surgery may help patients, but it is best to perform it in the inactive phase of the systemic disease [13]. While upper lid retraction is one of the most common symptoms requiring surgery in Graves’ orbitopathy, there is still no consensus regarding the best technique [2]. Surgical procedures that can achieve a predictable height and contour of the lid remain a challenge for orbit surgeons. Hence, various techniques have been reported, including anterior or posterior recession or resection of Müller’s muscle and/or the levator muscle or its aponeurosis, full thickness blepharotomy, levator lengthening by placement of a spacer that can be natural or synthetic, and marginal myotomy [4, 14]. Nonetheless, weakening or lengthening of the upper lid retractors is the basis of all techniques. In this study, we describe the results of a new surgical technique using lateral levator aponeurectomy via a transconjunctival approach in patients with upper eyelid retraction secondary to Graves’ orbitopathy.

## MATERIALS AND METHODS

This prospective clinical study of patients with moderate to severe eyelid retraction due to Graves’ orbitopathy was conducted at the Feiz Eye Hospital and the Department of Ophthalmology, Isfahan University of Medical Sciences, Isfahan, Iran from October 2015 to October 2016. All surgeries were performed by a single surgeon (AZ). Inclusion criteria were patients requiring surgical correction of upper eyelid retraction due to thyroid orbitopathy, patients with a stable thyroid state (as confirmed in laboratory and clinical experiments) for at least 6 months, and patients with no previous lid surgery. Patients who had previous lid surgery, age less than 18 years, or active or uncontrolled disease and patients who did not come for follow-up visits were excluded. The study protocol and ethical issues was approved by the institutional review board of Isfahan University of Medical Sciences. All participants underwent full examination prior to surgery. All the important harmful effects or unintended effects related to the surgery, as well as the novelty of the surgical technique, were completely explained to the patients. Written informed consent was obtained from all participants before the surgery. Patient age, sex, eye (right or left), and follow-up visits were recorded. In each visit, slit-lamp examination for exposure keratopathy and measurement of upper marginal reflex distance (MRD1) were recorded. Presence of exposure keratopathy was recorded as 'yes' or 'no'. MRD1 measurement was recorded in millimeters (mm) preoperatively and at each follow-up visit. Complications associated with the surgery were noted in each visit. Statistical analyses were performed using STATA version 11 (State Corp., College station, TX) and all considered P-values were two-sided.

**Figure 1:**
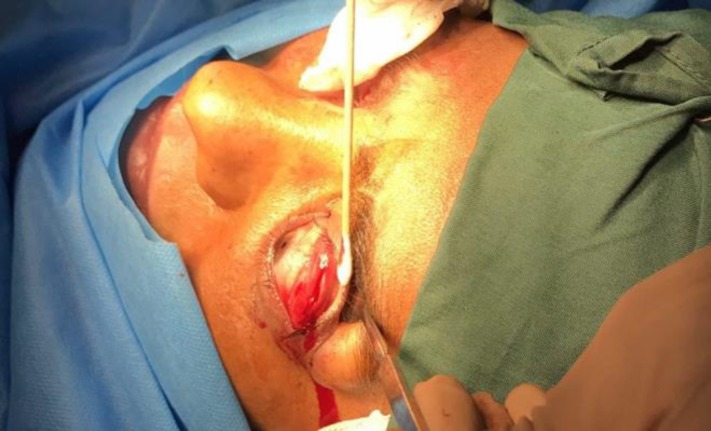
The First Step in Transconjunctival Lateral Levator Aponeurectomy Includes Lid Eversion and Incision Just Above the Tarsal Palate from the Lateral Margin

**Figure 2:**
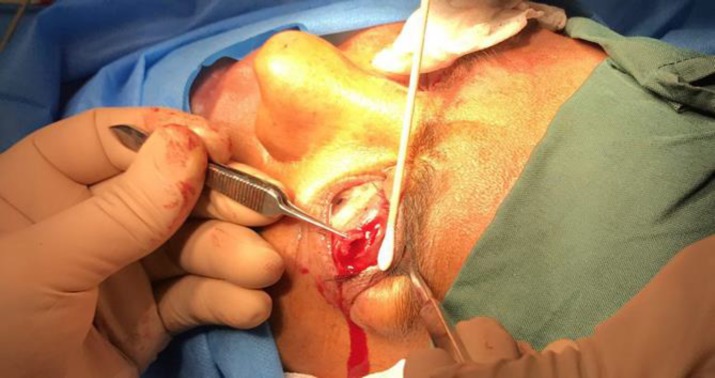
The Second Step in Transconjunctival Lateral Levator Aponeurectomy Includes the Dissection of the Conjunctiva and Müller’s Muscle and the Surgical Excision of the Levator Aponeurosis

Surgical Technique

Assessment of the ocular surface status and presence or absence of exposure keratopathy and measurement of MRD1 in seating position and in primary position were performed before the surgery. Surgery was performed under light sedation because of the need of a patient’s cooperation during the surgery, to reach symmetry between the two eyelids. The upper eyelid was anesthetized locally by injection of lidocaine 1% and 1:200000 adrenalin with a 30-gauge needle in subcutaneous tissue. The lid was everted by using a Desmarres lid retractor and the palpebral conjunctiva was exposed. The upper border of the tarsal palate was identified and an incision was made immediately above the tarsal palate from the lateral margin to the center of the lid at 1/2 to 1/3 of the upper lid length based on the intensity of lid retraction with a #15 knife (Fig 1).

**Figure 3:**
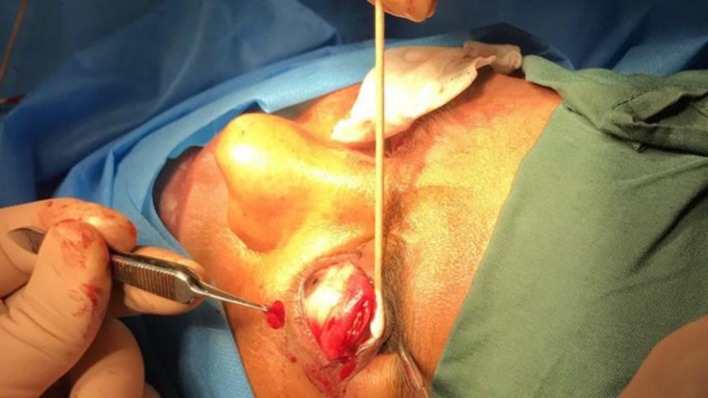
The Size of the Surgically excised Levator Aponeurosis during Transconjunctival Lateral Levator Aponeurectomy has the Same Length of the First Incision and 5 mm of Width

The conjunctiva and Müller’s muscle under the conjunctiva were dissected with a Westcott scissor to reach the levator muscle aponeurosis. After exposing the levator aponeurosis, it was surgically excised (Fig 2) with the same length of the first incision and 5 mm of width (Fig 3). The conjunctiva and Müller’s muscle were returned to their primary position. Patients were asked to attend the follow-up visits at 1 week and 3 and 6 months after the surgery. MRD1 was measured and recorded in each visit.

## RESULTS

This study included 16 eyes of 16 patients with Graves’ ophthalmopathy. All patients completed the study and were considered for final analysis. The age of the patients ranged between 29 and 60 years old (Table 1).

**Table 1:** Demographic Data of Patients with Graves’ Ophthalmopathy

	Values
Age (years)	45.06 ± 8.77
Age Range	29–60
Sex	
** Male**	4 (25)
** Female**	12 (75)
Eye	
** Right**	9 (56.2)
** Left**	7 (43.8)

Date presented as mean ± SD or No (%).

Pre-operative MRD1 was 6.00–11.00 mm (mean: 7.84 mm). One week after the surgery, MRD1 was 2.00–4.50 mm (mean: 3.59 mm) (Table 1). This difference was statistically significant (P < 0.001) (Table 2).

Three and six months after surgery, MRD1 was 4.50–6.00 mm (mean: 5.09 mm) and 4.50–6.00 mm (5.10 mm), respectively (P < 0.001). There were no major or persistent complications related to the surgery. None of the patients had exposure keratopathy before or after the surgery.

**Table 2:** Upper Marginal Reflex Distance (MRD1) Measurements Before and After the Surgery in Patients with Graves’ Ophthalmopathy

MRD1	Range (mm)	Mean (mm) ± SD
Before the surgery	6.00–11.00	7.84 ± 1.35
After the surgery		
**1 week**	2.00–4.50	3.59 ± 0.82
**3 months**	4.50–6.00	5.09 ± 0.52
**6 months**	4.50–6.00	5.10 ± 0.77

## DISCUSSION

Upper eyelid retraction is the chief morbidity of Graves’ ophthalmopathy, which can result in exposure keratopathy, proptosis, and cosmetic malformations. The aim of surgical correction of lid retraction is to prevent and treat keratopathy, relief ocular discomfort, and return the patient’s appearance [2]. Upper eyelid retraction is multifactorial and can be divided into two main categories: 1) anterior lamella involvement due to tissue loss or scaring that contract the skin and orbicularis oculi muscle, secondary to superficial injuries such as burns; and 2) levator mechanism shortening due to overactivity of Müller’s muscle with/without levator muscle. The latter type occurs most commonly secondary to Graves’ ophthalmopathy [7]. With the progression of the disease, levator and Müller’s muscles are affected by inflammatory processes, resulting in muscle overactivity. This stage leads to variable lid retraction without evidence of lid lag. Tissue changes and development of fibrosis in retractors of the lid terminate in restrictive patterns of lid retraction [8]. Eyelid retraction cannot be corrected completely by medication. The surgical techniques used for upper lid retraction are divided into the transconjunctival (posterior) approach to resect Müller’s muscle and the transcutaneous (anterior) approach through an eyelid crease to resect the levator muscle and its aponeurosis along with Müller’s muscle [7, 13]. The choice of surgical method depends on the degree of retraction and the experience of the surgeon.

In 1923, Goldstein described levator recession and in 1959, Berke suggested tenotomy for upper eyelid retraction [15]. Henderson described his surgical technique in 1965. This procedure consisted of a mullerectomy via conjunctival approach and then graded division of the levator aponeurosis fibers from the anterior surface of the tarsus plate. Henderson’s procedure is quick and does not need suturing, and because of the simple anatomy of the operation, it is still commonly used in the clinical setting. Olver and Fells reviewed Henderson’s procedure in the Moorfield Eye Hospital to identify the most appropriate patients for this procedure. They concluded that the procedure should be reserved for mild and symmetric (or near symmetrical) lid retractions that have no significant lateral flare. This surgery is done on superior and anterior tarsal plate. Therefore, it cannot correct lateral contour flare [5]. In 1983, Hurwitz and Rodgers reported the use of mullerectomy for mild retraction, mullerectomy combined with levator aponeurosis weakening for moderate retraction, and either mullerectomy and levator aponeurectomy or scleral graft implantation for severe retraction [16]. Contrary to the study of Hurwitz and Rodgers, in our study, levator aponeurectomy was performed using a transconjunctival approach. Therefore, in our patients, the technique was scales and had an acceptable clinical cosmetic outcome. We also did not use levator aponeurosis weakening or synthetic mesh as a spacer for surgical approach, which could have increased the risk of adhesion secondary to the procedure.

Small suggested a new surgical technique in 1988, called the proximal levator technique. In this procedure, the levator muscle proximal to Whitnall’s ligament is divided and then fixed with an adjustable suture [15, 17]. In this study, we reported a new surgical technique for correcting lid retraction in Graves’ ophthalmopathy. One week after the surgery, the mean MRD1 was reduced by 4.25 mm from the pre-operative measurement. We followed the patients 3 months after the surgery and the mean MRD1 was 5.09 mm, which means that the patient’s lid achieved the optimal position after 3 months. The change of the lid position and the significant differences of the post-operative MRDs raise the question that this change was secondary to normal recovery of edema of manipulated soft tissues or to surgical regression. For certainty of the stability of the surgical result, we decided to measure MRD1 6 months after the surgery. Mean MRD1 was 5.10 mm, which was similar to the measurement at 3 months after surgery.

The major limitations of this study were its cross-sectional nature and the small number of cases as well as absence of a control group.

In conclusion, we propose this simple, scar-less, quick surgical technique as a beneficial procedure for treating lid retraction stably.
